# The Significance of Selected Collagens and Their Connection with Relevant Extracellular Matrix Proteins in Bovine Early-Mid-Pregnancy and Parturition with and Without Retained Foetal Membranes

**DOI:** 10.3390/biom15020167

**Published:** 2025-01-23

**Authors:** Jacek Wawrzykowski, Monika Jamioł, Marta Kankofer

**Affiliations:** Department of Biochemistry, Faculty of Veterinary Medicine, University of Life Science in Lublin, Akademicka Street 12, 20-033 Lublin, Poland; jacek.wawrzykowski@up.lublin.pl (J.W.); monika.jamiol@up.lublin.pl (M.J.)

**Keywords:** collagen I, collagen IV, bovine placenta, placental attachment, placental retention

## Abstract

Appropriate placental structure and function assure foetal development, delivery of nutrients, and removal of waste. Collagens, as structural proteins, are crucial for the maintenance of placental growth and function. The aim of this study was to describe the profile of collagen 1 and 4 in the placental tissues of cows and to correlate it to previously described activities of collagenases and adhesive proteins. Placental samples were collected from pregnant cows in the slaughterhouse (2nd, 4th, and 6th month; n = 12) and during parturition after caesarean section. Samples taken during caesarean section were retrospectively divided into retained (R; n = 6) and not retained foetal membranes (NR; n = 6). Determinations were performed of maternal and foetal parts separately after tissue homogenisation. Supernatants were used for the determination of COL1 and COL4 concentrations by ELISA and WB analysis. Significant differences were detected between pregnancy months and parturient samples in COL1 concentrations and between retained and released foetal membranes. The concentrations of COL4 were higher in the foetal as compared to the maternal part of the placenta. Significant differences were detected between retained and released foetal membranes, and, similarly to Col1, values were lower in retained than released foetal membranes. WB analysis showed the presence of examined collagen molecules and their molecular weights. The analysis of collagen profile together with the enzymes of their degradation and other adhesive proteins (glycodelin, decorin, and thrombospondins) in bovine placenta either during pregnancy and parturition showed a close relationship. Either attachment or detachment of the maternal and foetal parts of the bovine placenta requires actions in concert between all these adhesive proteins under the influence of pregnancy hormones.

## 1. Introduction

One of the crucial aspects of placental structure is its stability despite remarkable temporal variability. The foetus relies on a properly functioning placenta to sustain its life from the early weeks of pregnancy until parturition. A number of drastic changes occur during delivery, and the placenta is expelled from the mother’s body in just a few hours. From a practical point of view, it is important to understand the mechanisms responsible for the functioning of the placenta during both pregnancy and parturition. Knowledge of the interdependence of certain physiological phenomena occurring during this period would make it possible to respond appropriately to abnormalities such as retained foetal membranes [1].

The bovine cotyledonary synepitheliochorial placenta consists of separate cotyledons with villous interdigitation between foetal villi and maternal crypts [2]. Implantation starts the process of pregnancy recognition and the formation of contact between mother and foetus. Equally important, and similarly poorly elucidated, is the process of placental attachment during placentation. It is supposed that discovering the mechanisms of placental attachment will allow us to understand placental detachment and its alterations, as well as the possibilities of preventing and successfully treating retained foetal membranes in cows. Jones et al. described changes at the glycosylation level within the layers of the mammalian placenta (horse, pig, cow, sheep, and human). They also indicated that the large variability in the amount and form of glycosylated derivatives within each layer is rather a component of changes occurring during placentation and pregnancy than their effect [3].

In ruminant species like cows, sheep, and goats, uterine collagens do not undergo the significant cyclic and pregnancy-related changes observed in species with highly invasive placentas (e.g., human) [4,5]. However, specific modifications in the composition and distribution of the extracellular matrix (ECM) have been documented during the oestrous cycle, early pregnancy, and near term [6].

During pregnancy and parturition, there is a significant reorganisation of the ECM. One of the possible pathways for successful delivery involves the degradation of collagen by specialised ECM collagenases. Several studies have been published indicating a correlation between the activity of these enzymes and the likelihood of placental retention in cows. Eiler and Hopkins tried to treat the retention of foetal membranes with injections of collagenases. The same authors pay attention to collagens and the whole ECM of the placentome as key players in the mechanisms of this syndrome [7]. Currently, the available literature confirms the participation of collagenases in the process of placental detachment [8,9].

Collagens are a group of protein molecules that possess a unique secondary structure resulting from posttranslational modifications. These modifications involve the hydroxylation of proline and lysine residues, converting them into hydroxyproline and hydroxylysine. This specific secondary structure enables collagens to function as structural proteins in connective tissue. Collagens are part of extracellular proteins and remain in close interplay with adhesive proteins in the bovine placenta. Thanks to extracellular remodelling, placentomes can increase their size and allow a better connection between mother and foetus.

Sarges et al. examined possible qualitative and quantitative differences in selected collagen molecules in placentomes collected postpartum from cows with and without retained foetal membranes but found no alterations. Authors suggested that not only might the total amount and distribution of these molecules be important, but the same could be true for their cooperation with other ECM compounds [10].

The balance between proteolytic enzymes and their activators and inhibitors is essential for the regular development of the placenta. In the case of collagens, this role is played by matrix metalloproteinases (MMPs) and their inhibitor TIMPs (tissue inhibitors of metalloproteinases). MMP-2 and MMP-9 are enzymes involved in trophoblast invasion by acting on collagen IV. The debate regarding the dominant gelatinase in this process continues. Some studies suggest that MMP-9 plays a more significant role, while others indicate that MMP-2′s action is more pronounced. In the human placenta, the expression of gelatinases in trophoblast cells varies depending on gestational age, with MMP-2 being the main gelatinase before 9 weeks of gestation and MMP-9 becoming more prominent thereafter. Furthermore, MMP-9 expression is higher in the third-trimester cytotrophoblast compared to the first-trimester trophoblast [11,12,13,14,15,16,17]. Maj and Kankofer indicated changes in the activity, and, therefore, the lack of active forms, of MMP-2 with masses of 64 and 60 kDa in the maternal and foetal parts of the bovine retained placenta, which could have influenced the hydrolysis of collagen and the proper release of foetal membranes in cows during parturition [8]. However, Walter and Boos did not observe differences in the distribution of MMP-2 and MMP-9 between cows with normal release of foetal membranes and cows with retained foetal membranes, which indicates a more complicated process of degradation of collagen structures than a simple change in enzyme concentrations [18]. The location of the activated form of MMP enzymes is relatively easy to demonstrate by zymography in gels containing gelatine. Wawrzykowski et al. showed that in the bovine placenta, the concentrations of MMP-3 and MMP-7 were significantly decreased during placental formation [19].

It is already known that some adhesive proteins influence collagen metabolism. An example of a protein closely related to collagen metabolism is decorin (DCN). DCN, a small leucine-rich proteoglycan, is known to play a crucial role in modulating collagen fibrillogenesis. By binding to collagen, DCN influences the diameter and spacing of collagen fibrils, contributing to the tissue’s structural integrity and mechanical properties. This interaction helps maintain the proper alignment and strength of the collagen matrix, which is essential for tissue function and resilience. Our previous studies demonstrated statistically significant changes in DCN concentration between samples from the foetal and maternal parts of the placenta during pregnancy in cows [20].

Thrombospondin-1 (TSP1, THBS-1) plays a role in collagen metabolism through close interactions with ECM, MMPs, and transforming growth factor-β1 (TGF-β1). THBS-1 and -2 have a binding region for collagens type I–V, allowing direct interaction between THBS and collagen [21]. Disturbances in the chain of connections between THBS, TGFβ1, and collagens may also be one of the causes of incorrect separation of placental tissues in cows during parturition. In our recent research, we demonstrated a statistically significant relationship between THBS and TGFβ1 concentrations and the release of foetal membranes after parturition in cows [22].

The aim of this study is to investigate the relationship between previously described adhesive proteins, collagenase activity, and other extracellular matrix proteins with the levels and structure of collagen proteins (types I and IV). This study focuses on these correlations during pregnancy, and on physiological and retained foetal membranes during parturition in cows.

## 2. Materials and Methods

### 2.1. Placental Tissue Samples

Early-pregnancy placental tissues were collected from healthy Holstein-Friesian cows, 4–6 years old, that underwent veterinary examination. The collection took place at a slaughterhouse during the 2nd (n = 5; 48–58 days of gestation), 4th (n = 4; 97–116 days of gestation), and 6th (n = 3; 157–161 days of gestation) month of pregnancy (total n = 12). The crown-rump length of the foetus was used to estimate the age of pregnancy. The uteri were consistently opened at the same location—from the middle part along the anti-mesometrial edge, and three placentomes near the incision site were carefully selected and removed. Parturient tissue samples were obtained from animals that underwent elective caesarean sections due to oversized foetuses. The sections were performed following standard veterinary practises, and the samples were collected under anaesthesia. Subsequently, the samples were retrospectively divided into two groups: the control group, consisting of not-retained foetal membranes (NR; n = 6), and the experimental group, consisting of retained foetal membranes (R; n = 6). Retained foetal membranes were defined as the absence of placental release within 8–12 h after delivery [23]. Care was taken to ensure that the samples were consistently collected from the same location in the uterus and from similar fragments of the placentome. The maternal and foetal parts of the placenta were manually separated during the sample collection process. Individual placental samples were homogenised in Ultra Turrax (Ikawerk, Janke, Kunkel, Staufen, Germany) on ice with 2 mL of PBS (0.1 mol/dm^3^, pH = 7.0), Triton X-100 (1%) and 20 µL protease inhibitor cocktail (Halt™ Protease Inhibitor Cocktail, Thermo Scientific™, 87785, Warszawa, Poland). Homogenates were centrifuged at 4 °C and 6500× *g* for 20 min. Obtained supernatants were aliquoted in vials and stored at −20 °C for subsequent Western blot and ELISA analyses. The procedure for obtaining the animal material after slaughter followed the previously established protocol by our research team [24,25,26,27]. All institutional and national guidelines for the care and use of animals were followed (EU Directive 2010/63/EU for animal experiments). Collecting samples during routine veterinary procedures does not require specific ethics permission (national ACT of 15 January 2015 on the Protection of Animals Used for Scientific or Educational Objectives).

In addition, the use of slaughtered material does not require the permission of the local ethics committee.

### 2.2. Western Blot

An equal amount of protein (20 µg per line) was separated using 7.5% SDS-PAGE. The separated proteins were then transferred onto PVDF membranes (Invitrogen, pb9320, Carlsbad, CA, USA) using the Trans-Blot^®^ Turbo™ Transfer System (25 V, 30 min, Bio-Rad, Warszawa, Poland), following the manufacturer’s instructions. After transfer, the membranes were blocked for 1 h in a TBST buffer (20 mmol/L Tris, 150 mmol/L NaCl, and 0.1% Tween 20; pH = 7.6) containing 4% low-fat milk. Subsequently, the membranes were incubated overnight at 4 °C with either 1.8 µg/mL COL1 (Invitrogen, PA1-26204, Carlsbad, CA, USA) or 1,5 µg/mL COL4 (Bioss. Bs-0806R, supplier ThermoScientific, Warszawa, Poland) antibodies. Following this, the membranes were incubated with AP-conjugated anti-rabbit antibodies (abbexa, ab6722, supplier Gentaur, Sopot, Poland) for 2 h at room temperature. For visualisation, 1-Step™ NBT/BCIP solution (ThermoScientific, 34042, Warszawa, Poland) was used. Staining for β-actin (ThermoScientific, PA1-183, Warszawa, Poland) was used as an internal control for WB. For transfer control, gels were stained with CBB (Roth, Roti^®^-Blue, A152.1, Zielona Góra, Poland). The signal obtained was analysed using Image Lab Software 4.0 (Bio-Rad, Warszawa, Poland) and normalised to the total protein in the same sample to account for variations in protein loading.

### 2.3. Determinations of COL1 and COL4

The COL1A1 assay was performed in tissue homogenates using the bovine collagen 1 alpha 1 ELISA kit (LSBio, LS-F11154-1, Shirley, MA, USA). Detection range: 1.56–100 ng/mL, sensitivity 0.55 ng/mL, intra-assay CV 5.3% and inter-assay CV 7.6%. The analysis of COL4A4 content was performed in tissue homogenates based on the bovine collagen alpha-4 chain ELISA kit (Jiaxing Korain Biotech (BT Labs), E0452Bo, supplier Gentaur, Sopot, Poland). Detection range: 5–320 ng/mL, sensitivity 2.71 ng/mL, intra-assay CV 8.0%, and inter-assay CV 10.0%. The analyses were carried out according to the manufacturer’s procedure. The obtained results were converted into the protein content in the sample (COL1A1 and COL4A4 pg per mg total protein).

### 2.4. Determination of Protein Content

Protein concentrations in homogenates were assessed using the DC protein assay method. The assay utilised the DC™ Protein Assay Kit II from Bio-Rad (#5000112), with bovine serum albumin (Merck, P02769, Warszawa, Poland) serving as the standard for calibration.

### 2.5. Statistical Analysis

Statistical analyses were performed using the Kruskal–Wallis [28] test followed by the Mann–Whitney U test [29]. For this purpose, the STATISTICA Version 13.0 software (StatSoft, Poland, TIBCO Software Inc., Palo Alto, CA, USA) was used. The results were presented as median ± quartiles. Significance was declared if *p* < 0.05.

## 3. Results

### 3.1. Western Blot

**Figure 1:**
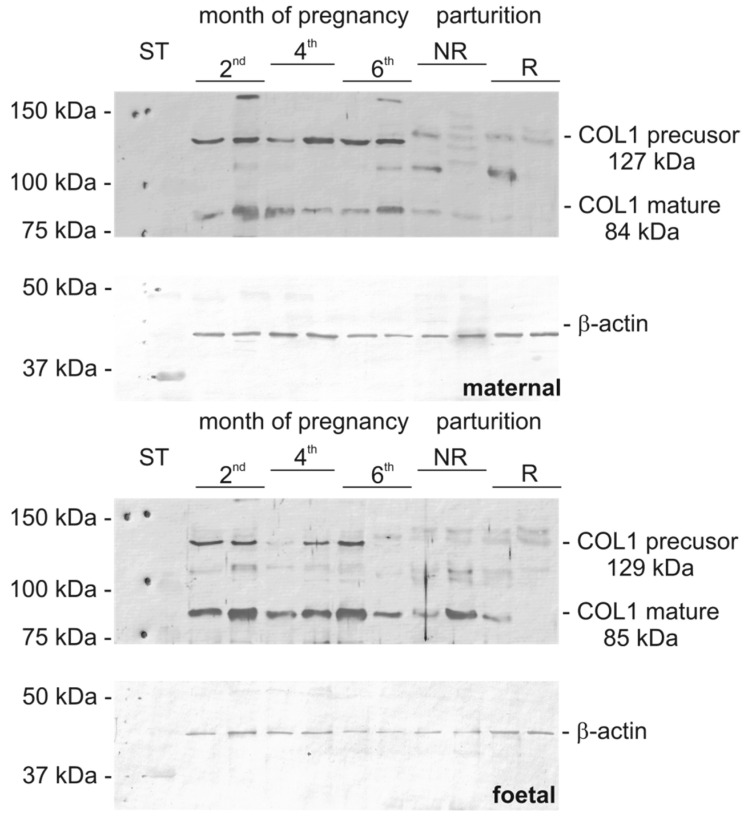
COL1A1 Western blot analysis in the placenta (maternal and foetal parts) of cows during pregnancy and parturition (2nd, 4th, and 6th month pregnancy period, NR, foetal membranes released up to 8–12 h; R, foetal membranes not released up to 8–12 h, ST—mass standard). Βeta-actin was used as loading control. The picture represents one of membranes with 2 randomly selected samples from each examined group. Original images can be found in supplementary materials Figure S2.

**Figure 2:**
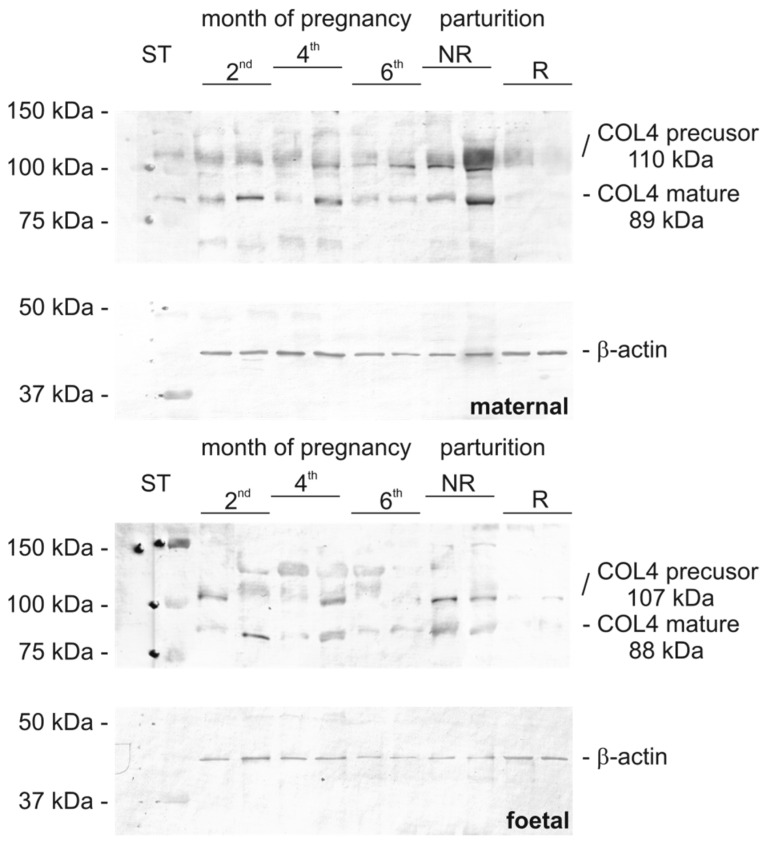
COL4A4 Western blot analysis in the placenta (maternal and foetal parts) of cows during pregnancy and parturition (2nd, 4th, and 6th month pregnancy period, NR, foetal membranes released up to 8–12 h; R, foetal membranes not released up to 8–12 h, ST—mass standard). Βeta-actin was used as loading control. The picture represents one of the membranes with 2 randomly selected samples from each examined group. Original images can be found in supplementary materials Figure S3.

Qualitative analysis by means of WB confirmed the presence of examined protein molecules in placental homogenates during pregnancy and parturition. Appropriate bands for COL1 were visible at 127.3 ± 1.7 kDa (precursor) and 84.2 ± 1.7 kDa (mature) in maternal part of the placenta, respectively (Figure 1), and 129.2 ± 2.7 kDa (precursor) and 85.0 ± 08 kDa (mature) in the foetal part. For COL4, visible bands were in the maternal part, with a precursor at 110.2 ± 1.3 kDa and fragments at 89.6 ± 2.6 kDa and in the foetal part at 107.0 ± 0.7 kDa and 88.5 ± 0.6 kDa, respectively (Figure 2). The composition of each gel covered 2 randomly selected representatives from each examined group (the same selected samples were used for all WB and controls gels). The whole gels are presented in Figure S1. The analysis of the optical density of the bands from the WB membrane are presented in Figures S4 and S5.

### 3.2. ELISA Determination of COL1A1 and COL4A4 Concentrations

**Figure 3:**
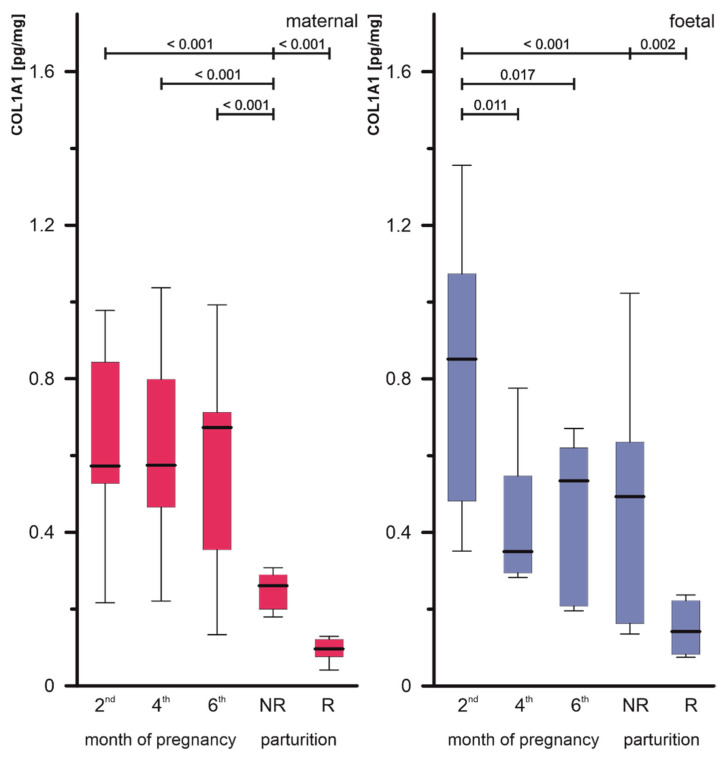
COL1A1 concentrations in the placentas (maternal and foetal parts) of cows during pregnancy (2nd, 4th and 6th month) and parturition (NR, foetal membranes released up to 8–12 h; R, foetal membranes not released up to 8–12 h). The horizontal line inside each box indicates the median. The box plot shades the lower and upper quartiles of the data. Whiskers represent the maximum and minimum values. The *p*-value from the Mann–Whitney U test, dependent variable: COL1A1 maternal and foetal, grouping variable: month; only statistically significant results were marked (*p* < 0.05).

**Figure 4:**
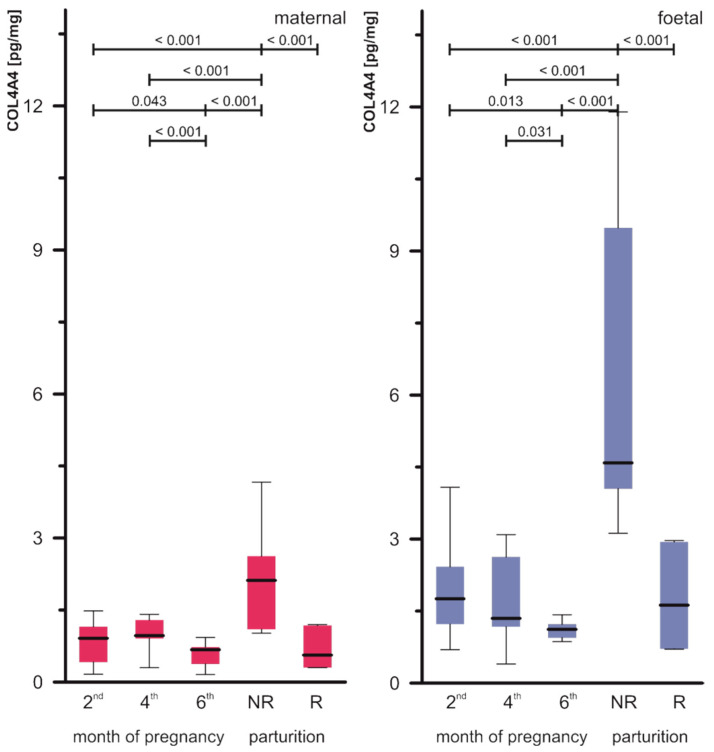
COL4A4 concentrations in the placentas (maternal and foetal parts) of cows during pregnancy (2nd, 4th, and 6th month) and parturition (NR, foetal membranes released up to 8–12 h; R, foetal membranes not released up to 8–12 h). The horizontal line inside each box indicates the median. The box plot shades the lower and upper quartiles of the data. Whiskers represent the maximum and minimum values. The *p*-value from the Mann–Whitney U test, dependent variable: COL4A4 maternal and foetal, grouping variable: month; only statistically significant results were marked (*p* < 0.05).

Quantitative analysis by means of ELISA assays expressed the profile of COL1 (Figure 3) and COL4 (Figure 4) in the placenta.

The concentrations of COL1 fluctuated between examined months of pregnancy in the foetal part of the placenta containing different types of cells. The highest value was detected in the 2nd month in the foetal part (0.871 ± 0.335 pg/mg protein) and it was statistically significantly higher than in both the 4th and 6th months. In the case of the foetal part, statistically significant differences were also found between the 2nd month of pregnancy and NR. In the maternal part of the placenta, a statistically significantly lower concentration of COL1 was found in NR compared to the studied months of pregnancy. Significant differences were noticed between retained and released samples of both examined parts of the placenta, expressing higher values in the last ones (NR maternal part 0.260 ± 0.043 pg/mg protein vs. R maternal part 0.096 ± 0.032 pg/mg protein and NR foetal part 0.493 ± 0.312 pg/mg protein vs. R foetal part 0.141 ± 0.069 pg/mg protein, respectively). Details of the statistical analysis of COL1A1 are presented in Figure S6.

The concentrations of COL4 expressed higher values in the foetal compared to the maternal part of the placenta. The values were statistically significantly lower in the 6th month of pregnancy compared to the 2nd and 4th months, both in the foetal and maternal parts. Similarly to COL1, values of COL4 were lower in the retained than in the not-retained placenta—the differences were significant both in the maternal and foetal parts (NR maternal part 2.188 ± 1.061 pg/mg protein vs. R maternal part 0.700 ± 0.356 pg/mg protein and NR foetal part 6.277 ± 3.119 pg/mg protein vs. R foetal part 1.730 ± 1.072 pg/mg protein). The concentration of COL4 in NR was statistically significantly higher compared to the studied months of pregnancy in the maternal part of the placenta. Similar relationships were demonstrated in the case of the foetal part, see details in Figure 4. Details of the statistical analysis of COL4A4 are presented in Figure S7.

## 4. Discussion

The present study describes the changes in collagen type I and IV concentrations and variations during early-mid-pregnancy, as well as during physiological parturition and when disturbed by retained foetal membranes. Obtained values were the highest in the 2nd month of pregnancy and lower in cows that retained foetal membranes in comparison to those that released them. Moreover, the results are discussed with the profile of selected adhesive proteins such as glycodelin, decorin, and thrombospondin.

The topic of these interrelationships in particular has caught our attention due to the fact that, for decades, scientists and practitioners have been trying to delve into aspects related to placental development, its proper construction, and anomalies in its functioning.

The placenta attaches to the uterine wall through a caruncular stalk. The interhaemal barrier is either epitheliochorial or synepitheliochorial, with trophoblast cells fusing with the uterine epithelium. The uterine wall has layers including the perimetrium, myometrium, and endometrium, which come into contact with intercotyledonar sections of the placenta. The endometrium consists of different layers, including the lamina epithelialis and lamina propria, which contain glandular openings and collagen fibres. Collagens are part of extracellular proteins and remain in a close interplay with adhesive proteins in the bovine placenta. The diverse functions of collagen are attributed to its classification into 28 different types, including fibrils (such as collagen types I and III), networks (collagen type VI), and beaded filaments (collagen type VI) [30]. These collagen molecules interact with six different receptors, namely integrins, discoidin domain receptors (DDR), leukocyte-associated immunoglobulin-like receptors (LAIR), glycoprotein VI (GPVI), the mannose-receptor family, and osteoclast-associated receptor (OSCAR) [31]. Thanks to extracellular remodelling, placentomes can increase their size and allow a better connection between mother and foetus. Microscopic and electron-microscopic studies have revealed that, in the collagen network of the matured pregnant cervix, there is a transformation from a tightly aligned arrangement of dense fibres to a partially degraded, loosely organised network characterised by shorter fibres. These shorter fibres can be extracted more readily from the collagen network [10].

Oefner et al. studied the role of COL4 during the menstrual cycle and the process of decidualization in women, where its alpha chains are selectively upregulated. Extravillous trophoblast cells express receptors for collagen and produce COL-IV both in laboratory conditions and within the body, leading to higher levels in the decidua basalis compared to the decidua parietalis. The three-dimensional network pattern of COL-IV was also discovered in the mesenchyme of placental villi. The NC1 domains of COL-IV alpha chains, known for regulating tumour cell migration, were found to be selectively expressed in the decidua basalis. This suggests that COL-IV is not only a structural protein but also plays a role in influencing the invasive behaviour of trophoblast cells at the implantation site in the placenta [32].

Interactions with adhesion proteins, enzymes involved in collagen breakdown, and their inhibitors are important in the regulation of collagen metabolism. The activity of specific enzymes can directly influence collagen content. There are several enzymes, previously mentioned in the introduction, that can break down collagen molecules depending on their collagen type. A review of the available literature indicates that MMP-14, MMP-2, and TIMP-2 are located in the compartment between placental membranes, and, therefore, by controlling collagen fibres, they can influence, on the one hand, placental fusion during implantation and, on the other hand, the timely release of foetal membranes in cattle (for details, see review: [9]). It also has been shown that glycodelin (Gd) concentrations decrease as pregnancy progresses (between the 2nd and 4th months of pregnancy). At the same time, MMP-2 concentrations remain at the same level during this period. During parturition, Gd and MMP-3 concentrations were significantly higher in the cows with retained foetal membranes (R group) compared to the cows that properly released them (NR group). Following the indicated above findings of Walter and Boos, the concentration of MMP-2 showed no significant differences between the examined groups (NR vs. R) [18,19].

The absolute amounts of collagens increase with ongoing pregnancy from 84 to 439 g before term [33] and decrease possibly a little around the time of and massively after parturition in ruminant species [33,34,35,36]. Collagen concentrations per gram of tissue, however, decrease slightly during pregnancy.

Boos et al. described the type I collagen layer as the main part of the connective tissue lying in the allantochorion, visible on the microscopic image as a relatively thick layer both under the uterine epithelium and on the periphery. During the development of bovine pregnancy, the increase in uterine mass is compensated primarily by the formation of placentomes. However, they did not observe a reduction in type 1 collagen during pregnancy [37]. This publication shows a quantitative change (concentration) in this type of collagen during the first months of bovine pregnancy. This is only partially consistent with the results reported by Yamada et al. [38], who examined changes in bovine COL1 and COL4 during the oestrous cycle and the early gestational stage (up to the 30th day of pregnancy) using immunofluorescence microscopy. In this study, type I and IV collagens were expressed in the caruncle throughout the study period except for the implantation period (from day 20 to 24) [38]. This reduced immunostaining at implantation sites was not confirmed by Boos et al. [37]. Inconsistent results regarding bovine collagens concern not only the pregnancy period, which, of course, requires further research, but also parturition. In our current study, we noted a clear difference in the amount of COL1 and COL4 in cows with normal release of foetal membranes compared to those with retained foetal membranes. Studies using immunohistochemical techniques have not shown any clear differences between the amount of collagen present just before delivery, at preterm caesarean section, or in the placenta at parturition [10,37]. However, as mentioned above, other studies indicate significant quantitative differences in type III collagen in cows with retained foetal membranes [39]. The existing discrepancies in the outcomes require further consideration to determine the involvement of collagens in the pathogenesis of retained foetal membranes. Taking into account previous reports [40], most probably the involvement is not direct, but rather takes place as a member of the cascade including MMPs, TIMPs, adhesive proteins, and sex hormones as regulators.

In addition to the prevailing studies using immunohistochemical techniques regarding the quantity and distribution of collagens, there are also transcriptomic reports. Guillomot et al. [41] tracked changes in the level of mRNA expression for COL1A1 in bovine placentas. During placentation, the genes were expressed mainly in the whole chorionic mesenchyme and caruncular stroma. An increase in mRNA expression for COL1A1 was observed during the first months of pregnancy (from day 18 to 62) in the caruncular tissues, i.e., starting from the differentiation of the extraembryonic mesoderm around implantation and the appearance of the foetal villi in the mesenchyme [41,42]. Indeed, in our study, we noticed a statistically higher concentration of COL1A1 in the 2nd month of pregnancy in cotyledon compared to the later months. However, the above-described mRNA expression results are difficult to interpret in relation to COL1 protein levels in the tissues presented here; they indicate the presence of additional regulatory mechanisms between gene expression and protein formation within the tissue. It is well-known that the presence of mRNA may not guarantee the synthesis of the corresponding protein.

Detailed 13C CPMAS NMR spectra analyses carried out by Dhital et al., despite the lack of chemical modifications within the collagen chains, showed increased mobility within some side chains of the amino acids Cγ-Val, Cγ-Glu, or Cα-Glu, Cα-Ser and decreased mobility in Cδ-Hyp or Cα-Leu in pregnant rats in relation to the control group. At the same time, it has been shown that changes in collagen fibres in the uteri of rats persist after delivery and during subsequent pregnancies. This poses major problems in tracking collagen changes within the uterus due to the need to select animals of the same age and gestation period [43]. Similarly, Peixoto et al. (2013) showed that the formation of collagen fibrils results from amino acids, such as proline and hydroxyproline. At the same time, based on statistical data, it was shown that a significant number of amino acids participate in the stabilisation of collagen fibrils; depending on the structure, this share can reach from 10 to even 15% of amino acids [44]. Changes in the structure of collagens consisting of triple-helixes may lead to their incorrect reading by discoidin domain receptors 1 and 2 (cell surface receptor tyrosine kinases, DDR1, and DDR2), receptors sensitive to GVMGFO (O is hydroxyproline) motifs present in collagens I, II, III and IV. An incorrect connection does not lead to autophosphorylation of the receptor and, consequently, to the transmission of the signal into the cell [45,46].

Serine kinases, along with THBS-1 and THBS-2, are linked to alterations in the ECM within collagen fibres and also affect the activities of MMPs and TIMPs. Thrombospondins (TSPs) are multidomain, extracellular glycoproteins that bind calcium and are conserved across species from sponges to humans. To investigate the biological consequences of TSP1 deficiency on ECM organisation, it is important to note that both THBS-1 and THBS-2 contain binding regions for type I–V collagens, facilitating direct interactions between THBS and collagen [47]. Rosini et al. demonstrated that THBS-1 directly influences collagen fibril formation in mice; when the genes responsible for encoding THBS-1 were inactive, collagen fibres in the skin exhibited significantly lower cross-linking levels compared to those of the control mice [48]. Bornstein et al. found that the ECM concentration of the MMP-2 is largely regulated by TSP2 and TSP1. An elevated MMP-2 concentration can result in decreased cell adhesion, irregular collagen fibre structure, and increased proliferation of endothelial cells [49]. In our previous study, we explored the relationship between THBS-1 and transforming growth factor beta1 (TGFβ1) concentrations during different stages of bovine pregnancy (second, fourth, and sixth months, normal parturition (NR), and in cases of parturition with foetal membrane retention (R)). The ELISA test revealed statistically significant differences (*p* < 0.05) in THBS1 concentrations (pg/mg protein) between the parturient samples, with higher levels observed in retained (R) foetal membranes compared to normal (NR). TGFβ1 concentrations (pg/mg protein) were significantly lower (*p* < 0.05) in retained foetal membranes compared to those that were normally released in the maternal part of the placenta (26.22 ± 7.53 in NR vs. 17.80 ± 5.01 in R). These changes indicate a correlation between these concentrations and alterations in collagen fibres during pregnancy and parturition.

DCN plays a crucial role in numerous cellular functions, such as collagen fibrillogenesis, wound healing, angiostasis, tumour development, and autophagy. Its functional diversity is due to the extensive range of interactions between DCN and various proteins, as well as its interaction with its singular glycosaminoglycan side chain. Structurally, DCN is highly conserved among various species, featuring a central domain that contains 12 leucine-rich repeats (LRR) and an N-terminal site for the attachment of a single glycosaminoglycan (GAG) chain, either chondroitin or dermatan sulphate. Importantly, the N-terminal GAG chain of DCN contributes to the organisation of neighbouring collagen fibrils. In particular, the dermatan sulphate chain binds to a different collagen molecule near the collagen fibre that interacts with the DCN protein core [50,51]. While the interaction between DCN and collagen type I is the most extensively studied, DCN also binds to other collagen types, including II, III, IV, V, VI, XII, and XIV, with a notably high affinity for collagen type VI. Related to collagen structure is the complex formed between DCN and matrilin-1, a protein involved in matrix assembly, whose relative, matrilin-3, has been linked to certain chondrodysplasias. This interaction speeds up collagen fibrillogenesis and influences the diameter of collagen fibres [52]. In vivo analyses have shown that collagen I gels in the presence of DCN are statistically more durable than those without it [53].

Our previous results on DCN concentration during pregnancy and delivery showed dynamic changes including its distribution within the maternal and foetal parts of the placenta. Both during pregnancy and physiological delivery, DCN levels were higher in the foetal part of the placenta compared to the maternal part [20]. We obtained similar relationship in this study, where the concentrations of both collagens I and IV were increased in the foetal part of the placenta compared to the maternal part. Moreover, adhesion studies using primary cultures of the uterine epithelial cells of pregnant cows showed a negative effect of DCN on cell adhesion to fibronectin in the 2nd month of pregnancy [25]. Interestingly, the results presented in this study indicated that the concentrations of collagens 1 and 4 were the highest in the 2nd month compared to at other time points during pregnancy. We speculate that the increase in collagen during placentation may result from the remodelling of ECM proteins leading to the increase in placentomes. DCN interacts with fibronectin, a protein involved in cell adhesion, migration, and differentiation, and with collagen, where DCN proteoglycan and its protein core can bind to fibronectin. Functionally, DCN inhibits fibroblast adhesion to the fibronectin substrate, and this interaction between DCN and fibronectin in vivo may lead to reduced cell adhesion to the ECM.

## 5. Conclusions

This study investigated the concentration of collagen types I and IV during the first two trimesters of pregnancy and at parturition in cattle. The interpretation of obtained results clearly shows that the changes in collagen concentrations and localisation are closely related to adhesive proteins of ECM. Their concert action and ECM remodelling, confirmed here by fluctuation of type I and IV collagen, might have a role in appropriate attachment, development and detachment of maternal and foetal part of the placenta in cows. The integration of knowledge about ECM metabolism can help in understanding the mechanisms responsible for placental development during all stages of pregnancy and parturition.

## Data Availability

Raw data including WB membranes and ELISA results are available by the first author on request.

## Supplementary Materials

The following supporting information can be downloaded at: https://www.mdpi.com/article/10.3390/biom15020167/s1, Original images of Western Blotting and statistical analysis can be found in supplementary materials.
