# Supplementary figures and images for: The Significance of Selected Collagens and Their Connection with Relevant Extracellular Matrix Proteins in Bovine Early-Mid-Pregnancy and Parturition with and Without Retained Foetal Membranes
Source: Biomolecules. 2025 Jan 23;15(2):167. doi: 10.3390/biom15020167 (PMC11852445; doi:10.3390/biom15020167)

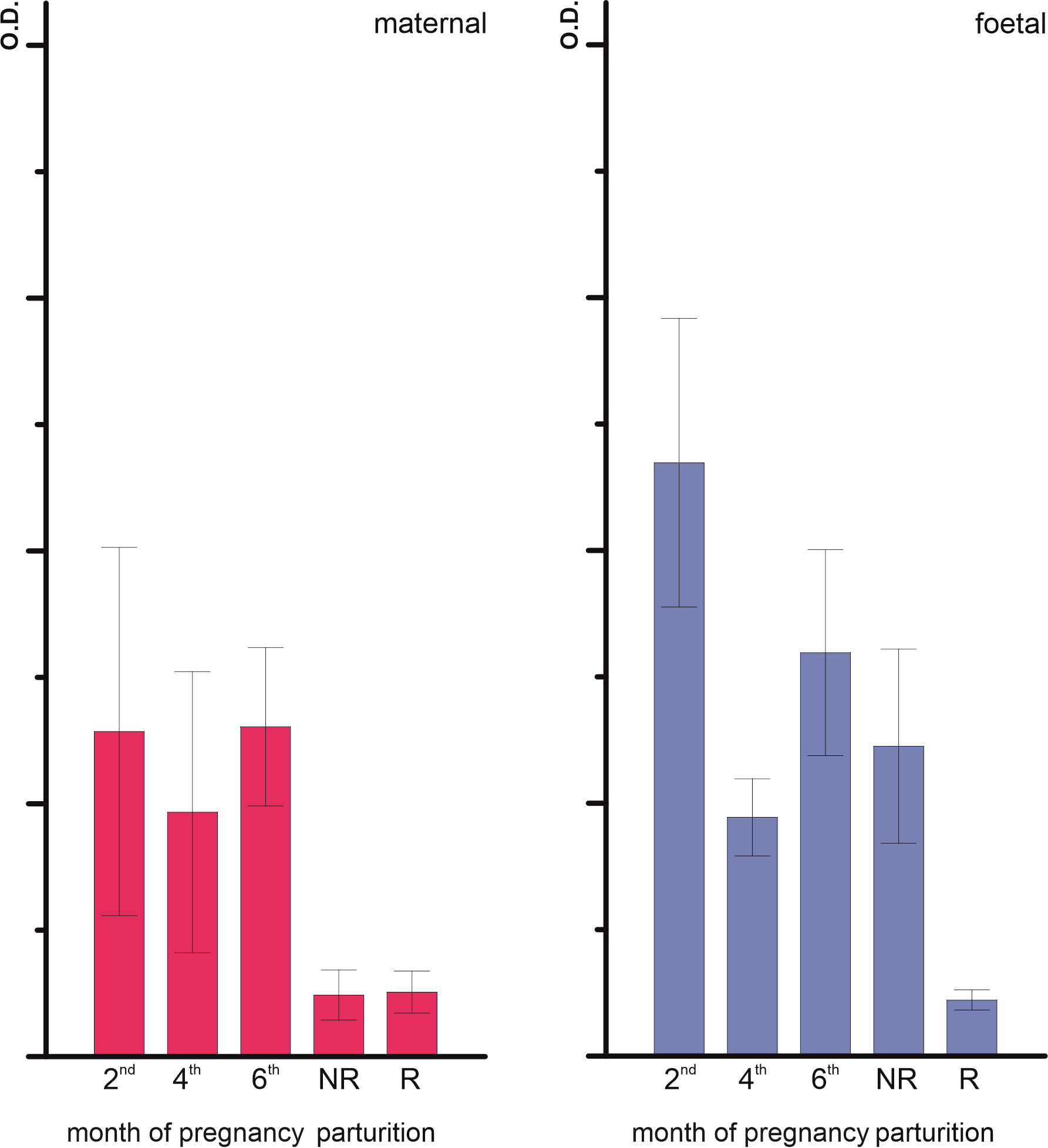

Supplement: Supplementary file 1 [file biomolecules-15-00167-s001.zip › biomolecules-3342099-supplementary/Figure S4. COL1_WB.tif]

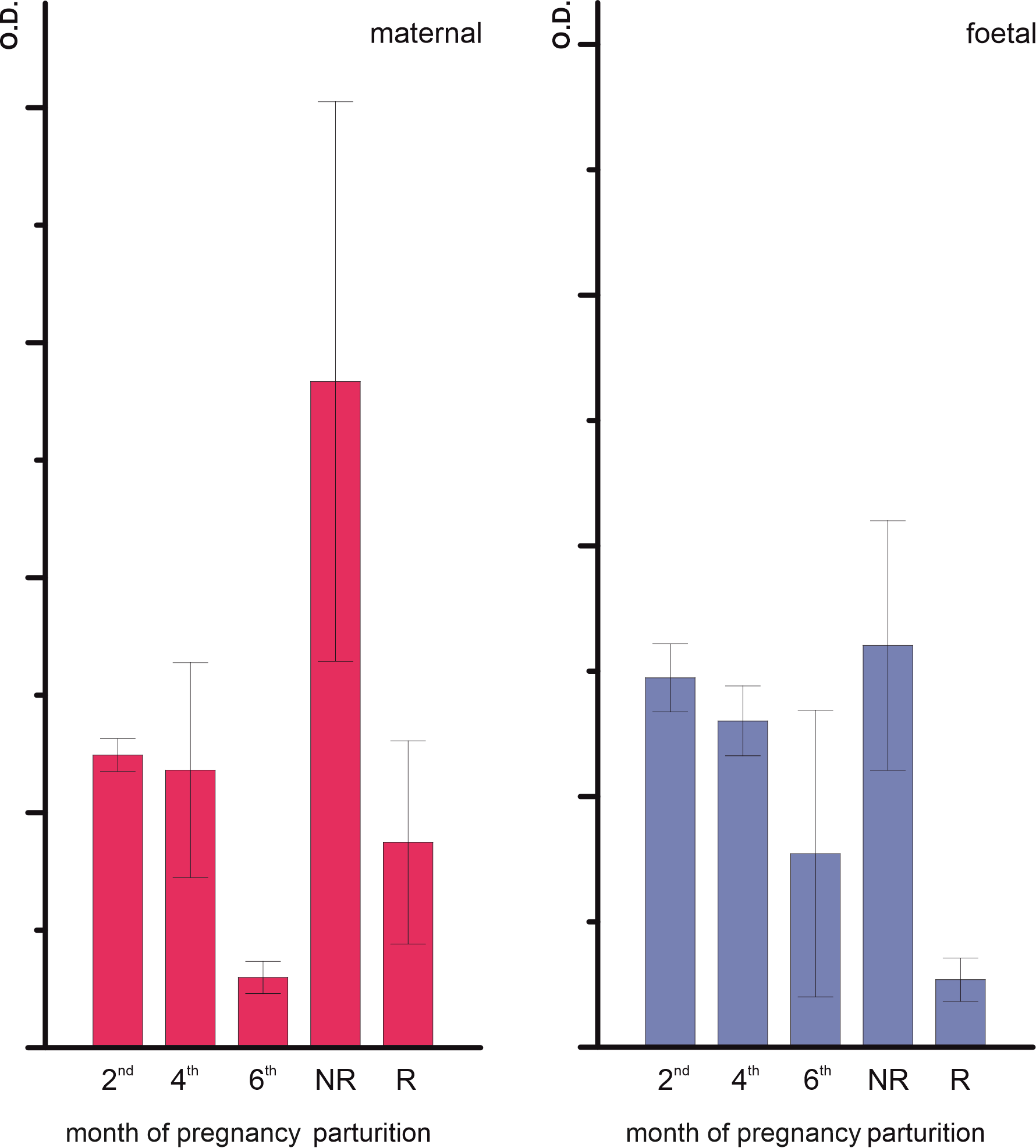

Supplement: Supplementary file 1 [file biomolecules-15-00167-s001.zip › biomolecules-3342099-supplementary/Figure S5. COL4_WB.tif]

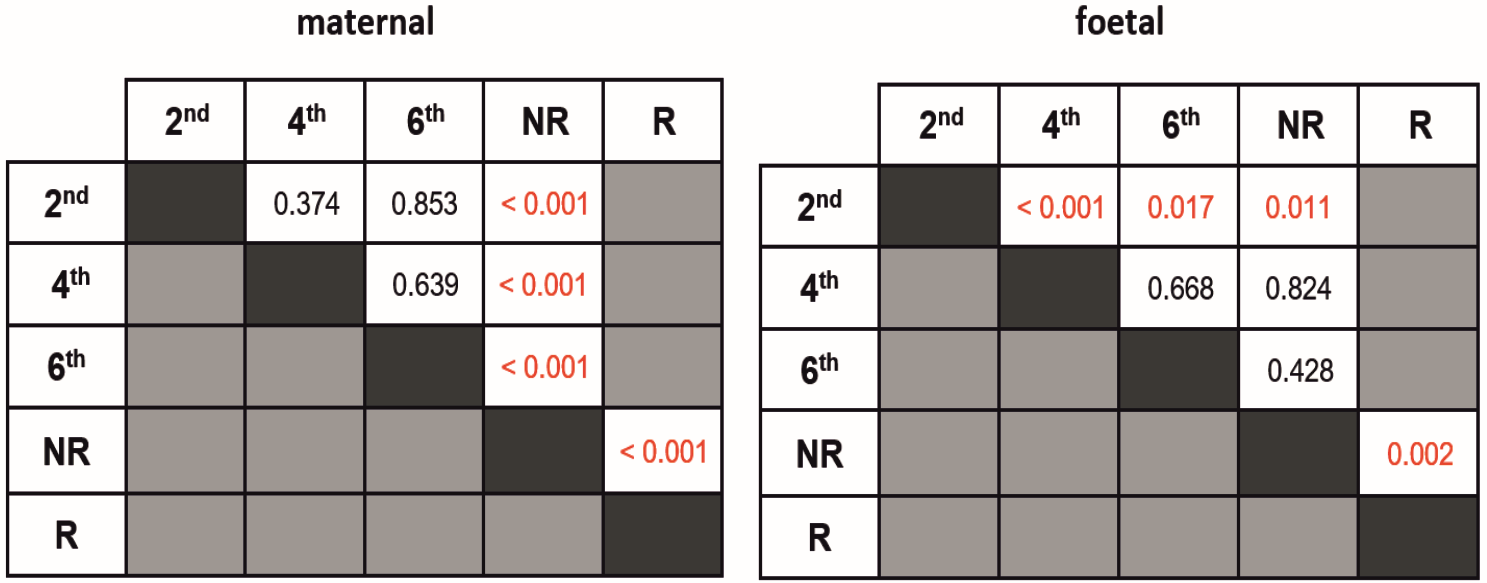

Supplement: Supplementary file 1 [file biomolecules-15-00167-s001.zip › biomolecules-3342099-supplementary/Figure S6. COL1A1 Mann-Whitney U.tif]

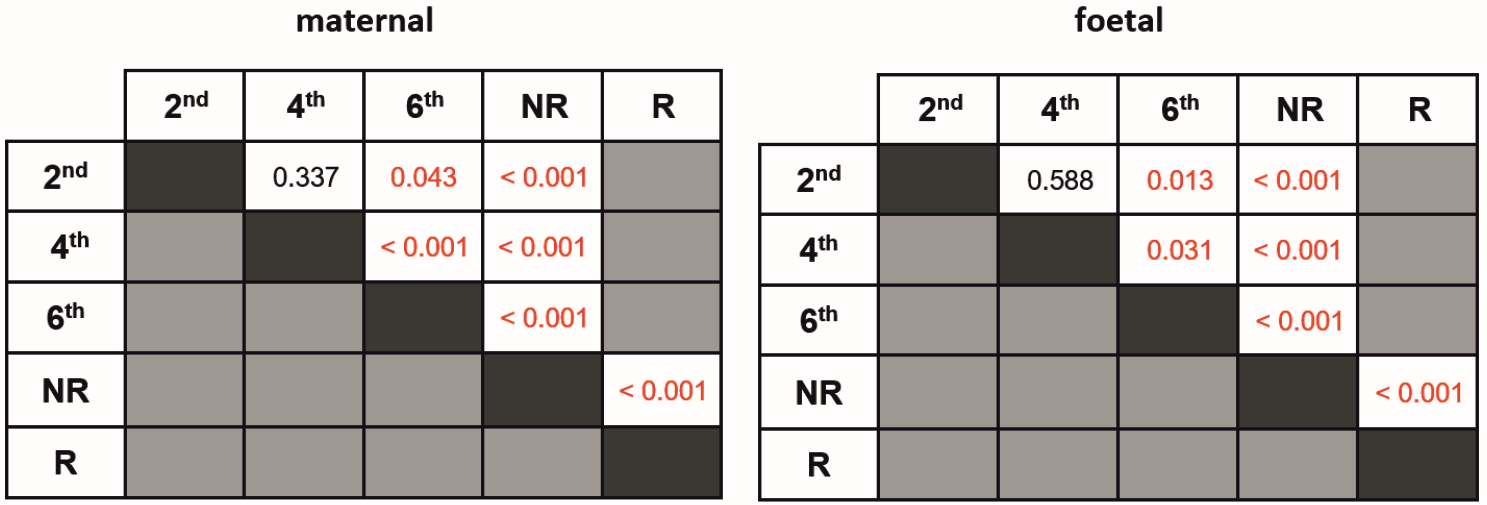

Supplement: Supplementary file 1 [file biomolecules-15-00167-s001.zip › biomolecules-3342099-supplementary/Figure S7. COL4A4 Mann-Whitney U.tif]
